# Women’s informed choice and satisfaction with immediate postpartum long-acting reversible contraception in Georgia

**DOI:** 10.1186/s40834-018-0073-x

**Published:** 2018-12-03

**Authors:** Carla L. DeSisto, Arden Handler, Sadia Haider, Rachel Caskey, Nadine Peacock, Melissa Kottke, Kristin Rankin

**Affiliations:** 1 University of Illinois at Chicago School of Public Health , 1603 W. Taylor St, Chicago, IL 60612 USA; 2 Department of Obstetrics and Gynecology, University of Chicago, 5841 S. Maryland Ave, Chicago, IL 60637 USA; 3 College of Medicine, University of Illinois at Chicago, 840 S. Wood St, Chicago, IL 60612 USA; 4 Department of Gynecology and Obstetrics, Jane Fonda Center, Emory University School of Medicine, 49 Jesse Hill Jr. Drive SE, Atlanta, GA 30303 USA

**Keywords:** Long-acting reversible contraception (LARC), Postpartum contraception, Informed choice, Medicaid policy

## Abstract

**Background:**

Several state Medicaid agencies have recently started reimbursing for long-acting reversible contraception (LARC) placement immediately postpartum. Women’s perspectives are critical for ensuring that this change increases access to LARC while empowering women to choose the method and timing of contraception that best meets their needs. We conducted a pilot study in Georgia, which recently changed its Medicaid reimbursement policy, to assess women’s informed choice and satisfaction with immediate postpartum LARC.

**Methods:**

We sampled all women with a live birth paid for by Georgia Medicaid during November 2015 through February 2017 who received an immediate postpartum LARC. We then used a one-to-one match to sample women who did not receive immediate postpartum LARC. Women were contacted via telephone for a 25–30 min interview regarding their knowledge, attitudes, and behaviors related to immediate postpartum LARC and their satisfaction with postpartum contraception. We calculated descriptive statistics and components of informed choice overall and by receipt of immediate postpartum LARC, using chi-square tests to calculate differences by group.

**Results:**

We approached 470 women and completed interviews with 51; 25 (49%) received immediate postpartum LARC (24 implants, 1 intrauterine device). Two-thirds reported their provider discussed the option of receiving immediate postpartum LARC during prenatal care, with over 90% reporting they received all the information they needed to make a decision. Most women believed the ideal time to begin using birth control postpartum is in the hospital immediately after delivery, although this differed significantly by women’s receipt of immediate postpartum LARC. Most women who received immediate postpartum LARC reported they are very or extremely happy with their device, although 40% also reported wanting their device removed at some point.

**Conclusions:**

Women on Medicaid in Georgia report making informed choices regarding immediate postpartum LARC. Among those who received immediate postpartum LARC, women report high levels of satisfaction.

## Background

Providing access to long-acting reversible contraception (LARC), which includes subdermal implants and intrauterine devices (IUDs), in the immediate postpartum period before hospital discharge has been recently promoted as a strategy for preventing unintended and rapid repeat pregnancies. This practice is considered safe by the Centers for Disease Control and Prevention [1] and the American Congress of Obstetricians and Gynecologists [2] and may be particularly convenient for women [3]. Several state Medicaid agencies have recently changed their policies to reimburse providers for LARC placement at the time of delivery [4], which may expand access given that Medicaid pays for approximately half of all deliveries across the nation [5] and Medicaid policies may later be adopted by private insurance plans.

There is much enthusiasm among providers and policy makers about the potential for immediate postpartum LARC to decrease unintended pregnancies and reduce public expenditures [3, 6]. However, there is also some concern that this enthusiasm, while often well-meaning, could lead to coercion and a general disregard for women’s informed choice and autonomy [7–9]. In light of past reproductive rights abuses against disadvantaged women in the U.S. [10], the recent documentation of women feeling coerced in their contraceptive decision making after undergoing abortion [11], and the fact that this initiative is specifically targeted to low-income women served by Medicaid, it is important to ensure that women’s choice and reproductive justice are central when developing and implementing these policies.

There is a dearth of evidence about women’s experiences with informed choice and satisfaction with immediate postpartum LARC. Women’s perspectives are critical for informing protocols to implement immediate postpartum LARC in a way that ensures increased knowledge and access while empowering women to choose the method and timing of contraception that best meets their needs. Therefore, we conducted a small pilot study in Georgia, which officially changed its Medicaid reimbursement policy in April 2014 [12]. Our objective was to assess the extent to which women choosing or not choosing immediate postpartum LARC are acting in alignment with their own preferences (“informed choice”) and in turn, whether or not they are satisfied with this decision.

## Methods

First, we sampled all women who had a live birth paid for by Georgia Medicaid during November 2015 through February 2017 and who received immediate postpartum LARC. Live births were identified using International Classification of Diseases, Ninth Revision (ICD-9) codes 650, V27.0, V27.2, V27.5, or V27.9 and ICD-10 codes O60.x-O77.x, O80.x, O82.x, Z37.0, Z37.2, Z37.5, or Z37.9 from Medicaid claims. Immediate postpartum LARC was identified if women had one of the following Healthcare Common Procedure Coding System (HCPCS) codes for a date of service that fell within the delivery hospitalization begin and end dates: J7300, J7301, J7302, or J7307. Women who experienced an infant death or permanent sterilization were excluded [ICD-9 codes: V25.2, V26.51, 656.4, V27.1, V27.3, V27.4, V27.6, V27.7, 66.21–66.29; ICD-10 codes: Z30.2, Z98.51, O36.4XX0, Z37.1, Z37.3, Z37.4, Z37.69, Z37.7, 0UL74ZZ, 0UL78ZZ, 0U574ZZ, 0U578ZZ, 0UL74CZ, 0UL74DZ, 0UL78DZ; HCPCS code: A4264; Current Procedural Terminology (CPT) codes: 58565, 58,600, 58,605, 58,615, 58,611, 58,670, 58,671, 58,340, 74,740]. Then, for each woman who received immediate postpartum LARC, we used Medicaid claims to sample an additional woman who had a live birth paid for by Georgia Medicaid but who did not receive immediate postpartum LARC. We used a 1-to-1 match on delivery month, age category (18–19, 20–24, 25–29, 30–34, and 35+ years), and delivery hospital to sample the women who did not receive immediate postpartum LARC. The total number of women sampled was 470.

Sampled women were mailed study information by partners at the Georgia Department of Community Health (DCH). Women were informed that researchers from the University of Illinois at Chicago would contact them via telephone for a study about postpartum contraception and given the option to opt out of participation via email or telephone. Women were informed that upon completion of an interview, they would receive a $25 gift card to their choice of Target or Walmart as a thank you for their time and participation.

Research assistants (RAs) attempted to contact the sampled women via telephone up to six times and left voice messages when possible. When contact was made with a woman, the RA briefly explained the study. If the woman was interested in participating, the RA would ask for her verbal consent to begin the interview or schedule the interview for another time.

We used mixed methods to assess informed choice and satisfaction. The interview guide consisted of mostly close-ended questions. However, we included some open-ended questions to allow women to explain their experiences and decision-making in their own words. Interview questions asked women about their most recent pregnancy and focused on three previously published components of informed choice, modified to address immediate postpartum LARC: knowledge/information provided, attitudes, and behaviors [13, 14]:

- Measures of knowledge/information provided: We asked women if they knew whether Georgia Medicaid paid for immediate postpartum LARC and whether it was possible for women to receive IUDs and contraceptive implants immediately postpartum. We also asked women to reflect on discussions with their medical providers about postpartum contraception during pregnancy and at the time of delivery.
- Measures of attitudes: We asked women about their ideal time to begin using contraception postpartum, the importance of different characteristics of contraceptive methods, and their future pregnancy plans.
- Measures of contraceptive behaviors: We asked women about their postpartum contraceptive practices and experiences with breastfeeding and attending a postpartum visit. We also asked an open-ended question about her process in deciding whether or not to obtain LARC immediately postpartum.

Additionally, we asked women about their satisfaction with their postpartum contraception and collected general demographic information. We did not use the word “LARC” in the interviews; instead we explained what IUDs and contraceptive implants are and asked about each device. For example, we said, “An intrauterine device or IUD is a small, plastic T-shaped device that is inserted in the uterus. Mirena and Skyla IUDs work for 3-5 years and the copper IUD, called Paraguard, works for up to 12 years.” Before asking questions related to IUDs or implants, we verified that the respondent understood what we were referring to and encouraged her to ask any clarifying questions she had.

All interviews were conducted in English. Interviews lasted approximately 25–30 min. Interviews were audio-recorded and the RAs entered data into a REDCap database [15]. Following completion of interviews, study data were transferred to a de-identified data set, which was analyzed in SAS version 9.4 (SAS Institute, Cary, NC). We calculated descriptive statistics of sample demographics and women’s knowledge, attitudes, and behaviors overall and by receipt of immediate postpartum LARC. Chi-square tests were used to calculate differences by group and statistical significance was assessed as *p* < .05.

The Institutional Review Board at the University of Illinois at Chicago approved this study (protocol #2015–0155).

## Results

Of the 470 women sampled, none emailed or called to opt out of participation. However, 12 were missing phone numbers and 163 phone numbers were disconnected or incorrect. Of the 295 women we were able to contact, 53 consented to participate (18.0% participation rate) and 51 completed interviews (96.2% completion rate). Three of the women who declined to participate did not speak English. The mean time from the infant’s birth to the interview was 35.5 weeks (standard deviation: 17.7 weeks; range: 14–83 weeks). Participants were an average age of 24.2 years. The majority completed high school or a General Equivalency Degree (GED), were unmarried, black, and had unintended pregnancies (Table 1). Participants had a similar age and delivery hospital distribution to the overall sampled population (*p* = 0.46 and *p* = 0.55, respectively, data not shown).

**Table 1:** Sample characteristics overall and by receipt of immediate postpartum long-acting reversible contraception

	Overall (*N* = 51) %	Received immediate postpartum LARC (*n* = 25) %	Did not receive immediate postpartum LARC (n = 26) %	Chi-square *p*-value
Maternal age (years)*				0.27
< 20	12.0	8.0	16.0	
20–24	44.0	56.0	32.0	
25–29	34.0	24.0	44.0	
≥ 30	10.0	12.0	8.0	
Race/Ethnicity				0.01
Black	66.7	88.0	46.2	
White	21.6	4.0	38.5	
Other	11.8	8.0	15.3	
Educational attainment				0.06
Less than high school diploma	11.8	16.0	7.7	
High school diploma or GED	49.0	32.0	65.4	
Some college or more	39.2	52.0	26.9	
Relationship status				0.14
Married	21.6	8.0	34.6	
Living with partner	33.3	40.0	26.9	
In a relationship not living with partner	11.8	12.0	11.6	
Single**	33.3	40.0	26.9	
Parity*				0.43
1	46.0	37.5	53.9	
2	28.0	29.2	26.9	
≥ 3	26.0	33.3	19.2	
Current health insurance				0.30
Medicaid	52.9	64.0	42.3	
Private insurance	5.9	4.0	7.7	
None	41.2	32.0	50.0	
Current employment status				0.20
Employed full-time	21.6	32.0	11.5	
Employed part-time	19.6	16.0	23.1	
Unemployed	58.8	42.0	65.4	
Pregnancy intention***				0.24
Intended	35.3	24.0	46.2	
Unintended	56.9	68.0	46.2	
Unsure	7.8	8.0	7.7	
Preterm birth (< 37 weeks gestation)	19.6	32.0	7.7	0.03
Delivery hospital				0.24
Hospital 1	68.6	80.0	57.7	
Hospital 2	25.5	16.0	34.6	
Hospital 3	5.9	4.0	7.7	

*1 missing

**“Single” includes women who reported being single and never married, separated, divorced, or widowed

***“Intended” includes women who reported that they wanted to be pregnant when they did or sooner; “unintended” includes women who reported that they wanted be pregnant later or that they did not want to be pregnant then or anytime in the future

About half the participants received immediate postpartum LARC (*n* = 25) and the other half did not (*n* = 26). Of those who received immediate postpartum LARC, one used an IUD and 24 used a contraceptive implant. Most demographic variables did not vary significantly by receipt of immediate postpartum LARC. However, women who received immediate postpartum LARC were more likely to be black and deliver preterm (< 37 weeks gestation) than women who did not receive immediate postpartum LARC. Women delivered at three different hospitals and lived in 12 different counties in Georgia, primarily representing the Atlanta and Savannah metropolitan areas (data not shown).

### Knowledge/information provided

The majority of participants reported discussing ways to delay pregnancy after delivery with their prenatal care provider (Table 2). Two-thirds reported their provider discussed the option of receiving immediate postpartum LARC during prenatal care and 71% reported being offered immediate postpartum LARC at the time of delivery. The majority reported receiving all the information necessary to make a decision.

**Table 2:** Knowledge provision and knowledge about availability of immediate postpartum long-acting reversible contraception, overall and by receipt

	Overall (N = 51) %	Received immediate postpartum LARC (n = 25) %	Did not receive immediate postpartum LARC (n = 26) %	Chi-square *p*-value
Discussed subsequent pregnancy prevention with provider during pregnancy?				0.67
Yes	90.2	92.0	88.5	
No	9.8	8.0	11.5	
During pregnancy, did provider offer option of immediate postpartum LARC?				0.12
Yes	66.7	76.0	57.7	
No	25.5	16.0	34.6	
Unsure	7.8	8.0	7.7	
During pregnancy, did provider give all the information needed to make a decision about immediate postpartum LARC?*				0.69
Yes	91.2	89.5	93.3	
No	8.8	10.5	6.7	
At time of delivery, did provider offer option of immediate postpartum LARC?				< .001
Yes	70.6	96.0	46.1	
No	29.4	4.0	53.9	
At time of delivery, did you have all the information needed to make a decision about immediate postpartum LARC?**				0.74
Yes	83.3	83.3	83.3	
No	13.9	12.5	16.7	
Unsure	2.8	4.2	0	
Does Medicaid in GA pay for immediate postpartum LARC?				0.32
Yes	86.3	92.0	80.8	
No	3.9	0	7.7	
Unsure	9.8	8.0	11.5	
Is it possible for a woman to receive IUD immediately postpartum?				0.10
Yes	51.0	60.0	42.3	
No	41.2	40.0	42.3	
Unsure	7.8	0	15.4	
Is it possible for a woman to receive implant immediately postpartum?				0.30
Yes	90.2	96.0	84.6	
No	5.9	4.0	7.7	
Unsure	3.9	0	7.7	

*N = 34; this question was only asked to women who said their provider offered them immediate postpartum LARC

**N = 36; this question was only asked to women who said they were offered immediate postpartum LARC at delivery

Among women who received immediate postpartum LARC, 92% reported signing a consent form at the hospital at the time of delivery, while 4% reported signing consent forms both during prenatal care and at the hospital during delivery; 4% did not remember when they signed a consent form.

The majority of women knew that Georgia Medicaid pays for immediate postpartum LARC (Table 2). Only about half knew it is possible to receive immediate postpartum IUDs, while the majority knew it is possible to receive immediate postpartum contraceptive implants.

### Attitudes

Most participants believed that the ideal time to begin using birth control postpartum is in the hospital immediately after delivery, although this differed significantly by women’s receipt of immediate postpartum LARC (Table 3). The majority reported that it was very important to have a birth control method they do not have to think about, they do not have to take every day, is very effective, is safe, and has very few side effects. About one-third reported not wanting to get pregnant again at any time in the future, while most others reported intention to be pregnant again in two or more years.

**Table 3:** Contraceptive attitudes overall and by receipt of immediate postpartum long-acting reversible contraception

	Overall (N = 51) %	Received immediate postpartum LARC (n = 25) %	Did not receive immediate postpartum LARC (n = 26) %	Chi-square *p*-value
Ideal time to begin using birth control postpartum				0.03
In the hospital after delivery	62.7	84.0	42.3	
After delivery hospitalization but before postpartum visit	2.0	0	3.9	
At the 4–6 week postpartum visit	21.6	8.0	34.6	
After the postpartum visit	9.8	8.0	11.5	
Unsure	3.9	0	7.7	
Importance of having birth control method you do not have to think about regularly				0.81
Very important	68.6	72.0	65.4	
Somewhat important	25.5	24.0	26.9	
Not important	5.9	4.0	7.7	
Importance of having birth control method you do not have to take every day				0.47
Very important	66.7	76.0	57.7	
Somewhat important	19.6	16.0	23.1	
Not important	11.8	8.0	15.4	
Unsure	2.0	0	3.8	
Importance of having very effective birth control method				0.17
Very important	84.3	92.0	76.9	
Somewhat important	9.8	4.0	15.4	
Not important	3.9	0	7.7	
Unsure	2.0	4.0	0	
Importance of having safe birth control method				0.61
Very important	94.1	96.0	92.3	
Somewhat important	3.9	4.0	3.9	
Not important	2.0	0	3.9	
Importance of birth control method having few side effects				0.22
Very important	94.1	92.0	96.1	
Somewhat important	3.9	8.0	0	
Not important	2.0	0	3.9	
Future pregnancy plans				0.02
Do not want to get pregnant at any time in the future	33.3	32.0	34.6	
Want to be pregnant again in 6 months to 2 years	17.6	4.0	30.8	
Want to be pregnant again in 2+ years	45.1	60.0	30.8	
Unsure	3.9	4.0	3.8	

### Behaviors

Women reported using a range of contraceptives between delivery and the interview, with the majority using condoms either alone or in combination with another method (Table 4). Most women reported initiating breastfeeding, and duration did not vary by receipt of immediate postpartum LARC. The majority of women reported attending a postpartum visit.

**Table 4:** Contraceptive and postpartum behaviors overall and by receipt of immediate postpartum long-acting reversible contraception

	Overall (N = 51) %	Received immediate postpartum LARC (n = 25) %	Did not receive immediate postpartum LARC (n = 26) %	Chi-square *p*-value
Contraceptives used since delivery*				
Contraceptive implant	51.0	96.0	7.7	< .01
IUD	7.8	4.0	11.5	0.32
Sterilization	3.9	0	7.7	0.16
Oral contraceptive pill	13.7	4.0	23.1	0.05
Depo-Provera shot	17.6	4.0	30.8	0.01
Condoms	60.8	56.0	65.4	0.49
Emergency contraception	2.0	0	3.8	0.32
None	5.9	0	11.5	0.08
Other	5.9	4.0	7.7	0.58
Breastfeeding				0.29
Did not breastfeed	21.6	24.0	19.2	
≤ 1 month	27.5	20.0	34.6	
2–3 months	25.5	26.0	15.4	
≥ 4 months	25.5	20.0	30.8	
Postpartum visit attendance				0.57
Yes	80.4	80.0	80.8	
No	17.6	20.0	15.4	
Unsure	2.0	0	3.8	

*Women could select more than one option

### Decision-making

Few women reported feeling pressured to make a particular decision about immediate postpartum LARC. In response to our open-ended questions, women who received immediate postpartum LARC described the convenience of receiving contraception at the time of delivery, their desire for a long-acting method, and their past experiences with other contraceptive methods. The exceptions were two women who felt like their doctor and father were pressuring them to receive immediate postpartum implants.

Most women who did not receive immediate postpartum LARC desired another contraceptive method instead. When given a list of possible reasons they did not want immediate postpartum LARC, the most common responses were concerns about side effects and disliking the idea of a foreign object in their bodies (Table 5). However, of the 26 women who did not receive immediate postpartum LARC, three reported wanting immediate postpartum IUD (*n* = 1) or implant (*n* = 2). These women were unable to receive their desired devices due to complications during delivery, lack of stocked device at the hospital, and not realizing it was an option until after hospital discharge, respectively.

**Table 5:** Reasons for not wanting/choosing immediate postpartum LARC (*n* = 20)

Reason	%
Had concerns about side effects	75.0
Did not like the idea of a foreign object	70.0
Did not like the idea of hormones in body	60.0
Did not like that it cannot be removed by self	50.0
Afraid it was going to hurt	40.0
Preferred another kind of birth control	40.0
Wanted to wait until postpartum visit	40.0
Worried that LARC would make breastfeeding harder	35.0
Partner did not want her to get LARC	30.0
Did not know could get one right after delivery, before leaving the hospital	30.0
Did not have the money to pay for it	25.0
Did not know enough about LARCs	20.0
Want to get pregnant again within a year	20.0
Other family members or friends did not want her to get LARC	15.0

### Satisfaction

Most women who received immediate postpartum LARC reported they are very (40%) or extremely (32%) happy with their device. Of those who received immediate postpartum LARC, 80% reported they would recommend it to a friend. None of the women using IUDs (1 immediately postpartum, 3 interval placements) experienced a device expulsion.

Of the 30 women who received a LARC (26 implants, 4 IUDs), either immediately postpartum or later in the postpartum period, 13 (43.3%) said they wanted the device removed at one point; 10 of these had immediate postpartum implants. Two women had their implants removed by the time of the interview (1 immediate postpartum placement and 1 interval placement). Among the remaining 11 women, three reported they were planning to have their immediate postpartum implants removed soon due to side effects. The other women said they were waiting to see if their side effects became more tolerable or they felt it was worth keeping their device to prevent pregnancy.

## Discussion

To our knowledge, this is the first study to examine postpartum women’s experiences with informed choice and satisfaction with immediate postpartum LARC. Our conceptual framework for informed choice focused on women’s knowledge, attitudes, and behaviors, with the understanding that informed choice requires knowledge plus behaviors that are consistent with attitudes [13, 14]. We piloted the use of a novel population-based sampling technique in partnership with Georgia DCH to interview women who did and did not receive immediate postpartum LARC in two large metropolitan areas in Georgia. Women were well-informed about the fact that Georgia Medicaid reimburses for immediate postpartum LARC, reported receiving enough information to make a decision about using immediate postpartum LARC, and were generally satisfied with their experiences with LARC.

It was surprising that 90% of women in our study knew it was possible to receive an immediate postpartum implant, but about 41% did not think it was possible to receive an immediate postpartum IUD. While this aligns with the fact that the majority of women who received immediate postpartum LARC in our study obtained a contraceptive implant, it is contrary to other published studies reporting that IUDs are used more commonly than implants immediately postpartum [9, 16]. However, a Georgia study found that among early-adopting hospitals that placed immediate postpartum LARC, one was offering IUDs, one was offering implants, and two were offering both devices [17]. It is unclear if all three delivery hospitals represented in our study offered both IUDs and implants, but our results likely reflect the immediate postpartum LARC implementation process at the hospital level.

Most participants reported satisfaction with their devices, although more than two-thirds reported wanting the device removed at some point. Our results are consistent with previous studies about women’s satisfaction and removal rates of immediate postpartum contraceptive implants [18]. A recently published study found that women had a high degree of satisfaction with immediate postpartum IUD insertion, but were unable to understand or recall the consent process [19]. In our study, we asked women if they recalled signing a consent form and if they received enough information to make a decision about immediate postpartum LARC. While our results are promising, the number of women desiring device removal at some point may reflect an ineffective informed consent process, specifically regarding potential side effects of implants. However, it is unclear if women attributed common postpartum symptoms, such as bleeding and cramping, to implants, which have been shown to produce these side effects in some women [20].

There has been concern that barriers to removal may inhibit uptake of LARC methods. Several women in our study reported that they did not want or choose immediate postpartum LARC because they did not like that they cannot remove the device themselves. Some women may encounter challenges to getting a LARC device removed when they desire, including resistance from providers and cost barriers [21]. Although Georgia Medicaid does not have any policies that may add barriers to LARC removal the way a few other states do [22], 41% of the women in our study were uninsured by the time of the interview. Additional examination of the data showed that of the 11 women who reported wanting their LARC removed but who had not had a removal by the time of the interview, 5 were uninsured and the other 6 remained on Medicaid. We did not specifically ask women if insurance affected their decision-making about LARC removal, but none of the women mentioned this in response to open-ended questions about their experiences. However, in order to ensure women’s reproductive autonomy, it is important that providers and policy-makers do not impede women’s decisions to have their LARC devices removed. Women receiving immediate postpartum LARC in our study were more likely to be black and to deliver preterm compared to women not receiving immediate postpartum LARC. Consistent with national data, black women were more likely to deliver preterm than other racial/ethnic groups in our study (data not shown). Further examination of the data revealed that the proportion of women reporting that immediate postpartum LARC was offered to them varied significantly by hospital of delivery. Since black women in our study more often delivered at the hospital most likely to offer immediate postpartum LARC (data not shown), the racial difference in receipt of immediate postpartum LARC is likely a result of the demographic distribution in Georgia and the uneven implementation of immediate postpartum LARC in hospitals around the state at the time.

Public health concerns about immediate postpartum LARC have included potential effects on breastfeeding and women’s attendance at the 4–6 week postpartum visit [23]. In our study, neither of these outcomes varied by immediate postpartum LARC receipt. Similarly, there has been concern that women who use LARCs may be more prone to sexually transmitted infections because they may not feel the need to use barrier methods. However, more than half the women in our study, regardless of immediate postpartum LARC receipt, reported using condoms.

This study has several limitations. First, the low participation rate led to a relatively small sample with unknown generalizability. Women were notified about the study via mail and contacted via telephone based on information on file with Medicaid. Many women were uninsured by the time of the interview, which likely explains why Medicaid did not have their updated information. However, women we could not get in touch with may be particularly mobile or prone to changes in phone numbers, and may be different than the women we surveyed. Further, although we tried to contact women at various times, and asked women for a preferred time to call them back, nearly 60% of the women in our study were unemployed at the time of the interview, suggesting that our sample under-represents women in the workforce who may not have had time to participate. Few women in our study were Hispanic, Asian, or other races/ethnicities. Of all live births in Georgia during 2013–2015, 13.3% were Hispanic, 0.1% were American Indian/Alaska Native, and 4.4% were Asian/Pacific Islander [24]. However, the racial/ethnic breakdown of Medicaid-paid births in Georgia is unclear. We only interviewed women who spoke English. Although only three women are known to be excluded due to language, it is unknown how many women did not answer the phone or return our calls because they do not speak English. Also, due to the pattern of immediate postpartum LARC implementation in Georgia to date, women were only drawn from three hospitals. Therefore, despite the population-based sampling design, unevenness of implementation across the state and the low participation rate among women may have affected the generalizability of our results. Additionally, the time between delivery and interview ranged from 14 weeks to 20 months, which may have introduced recall issues, especially related to the counseling and decision-making process.

## Conclusions

In conclusion, women on Medicaid in Georgia report making informed choices regarding immediate postpartum LARC. These findings about women’s perspectives and desires for immediate postpartum LARC support continued efforts to increase immediate postpartum LARC access, while ensuring informed consent among all women. Ultimately, providing access to all methods of contraception at multiple time points during the postpartum period is the most effective approach to meeting women’s contraceptive needs.

## Abbreviations

CPT: Current Procedural Terminology
DCH: Department of Community Health
GED: General Equivalency Degree
HCPCS: Healthcare Common Procedure Coding System
ICD: International Classification of Diseases
IUD: Intrauterine device
LARC: Long-acting reversible contraception
RA: Research assistant
